# Experimental and Simulation Studies of Temperature Effect on Thermophysical Properties of Graphene-Based Polylactic Acid

**DOI:** 10.3390/ma15030986

**Published:** 2022-01-27

**Authors:** Giovanni Spinelli, Rosella Guarini, Rumiana Kotsilkova, Todor Batakliev, Evgeni Ivanov, Vittorio Romano

**Affiliations:** 1 Faculty of Transport Sciences and Technologies, University of Study “Giustino Fortunato”, Via Raffaele Delcogliano 12, 82100 Benevento, Italy; 2 Open Laboratory on Experimental Micro and Nano Mechanics, Institute of Mechanics, Bulgarian Academy of Sciences, Acad. G. Bonchev Str., Block 4, 1113 Sofia, Bulgaria; rgrosagi@gmail.com (R.G.); kotsilkova@yahoo.com (R.K.); todorbat@gmail.com (T.B.), ivanov_evgeni@yahoo.com (E.I.); 3 Research and Development of Nanomaterials and Nanotechnologies, NanoTech Lab Ltd., Acad. G. Bonchev Str., Block 4, 1113 Sofia, Bulgaria; 4 Department of Industrial Engineering, University of Salerno, Via Giovanni Paolo II, 84084 Fisciano, Italy; vromano@unisa.it

**Keywords:** biodegradable polymers, graphene nanoplatelets, nanocomposites, thermal transport properties, thermophysical properties, multiphysics simulations

## Abstract

Overheating effect is a crucial issue in different fields. Thermally conductive polymer-based heat sinks, with lightweight and moldability features as well as high-performance and reliability, are promising candidates in solving such inconvenience. The present work deals with the experimental evaluation of the temperature effect on the thermophysical properties of nanocomposites made with polylactic acid (PLA) reinforced with two different weight percentages (3 and 6 wt%) of graphene nanoplatelets (GNPs). Thermal conductivity and diffusivity, as well as specific heat capacity, are measured in the temperature range between 298.15 and 373.15 K. At the lowest temperature (298.15 K), an improvement of 171% is observed for the thermal conductivity compared to the unfilled matrix due to the addition of 6 wt% of GNPs, whereas at the highest temperature (372.15 K) such enhancement is about of 155%. Some of the most important mechanical properties, mainly hardness and Young’s modulus, maximum flexural stress, and tangent modulus of elasticity, are also evaluated as a function of the GNPs content. Moreover, thermal simulations based on the finite element method (FEM) have been carried out to predict the thermal performance of the investigated nanocomposites in view of their practical use in thermal applications. Results seem quite suitable in this regard.

## 1. Introduction

Polymers are largely present in our daily life and adopted for different industrial applications because of their low density and, therefore, light weight as well as low cost, remarkable chemical stability, and excellent processability [1]. However, a defining characteristic of polymer materials is their poor thermal properties (due to the random morphology of molecules chains), which represent a technological barrier in heat transfer problems for their practical use, such as heat sinks and electronic packaging [2]. In fact, as electronic devices become more and more miniaturized and designed to work at increasingly operating frequencies and current densities, their thermal management is a critical issue. Hence, efficient heat dissipation is mandatory for their best performance, reliability, and service life. Therefore, the possibility of enhancing the thermophysical features of polymers while preserving their aforementioned characteristics represents an attractive solution for such specific applications, and, over the last two decades, this research topic has become very active both at the industrial and academy level [3,4,5]. A scientifically recognized method of improving the thermal conductivity of polymers is by engineering them with the introduction of thermally conductive fillers, especially the carbon-based ones such as nanotubes (CNTs) and graphene nanoplatelets (GNPs). The latter type of filler, at suitable amounts and dispersion state, creates a 3-dimensional network capable of favoring dynamics of phonon transport, which results in an enhancing thermal property of the resulting materials [6,7].

It is important to point out that, although GNPs have excellent thermal properties with a conductivity of about 3000 Wm^−1^K^−1^ in the parallel to planes direction, such value is absolutely not achievable for composite structures. For nanocomposite materials, the overall thermal response is the result of a combination of several factors such as filler concentration, its shape, size, and orientation, as well as the matrix/filler interface or even the fabrication methods [8,9,10]. This means that, despite the latest scientific findings in the field of design, development, and optimization of such innovative materials, the achieved results are still far from those expected, and therefore, it is still not possible to fully benefit from the potential of nanotechnologies. Many research efforts are currently devoted to experimental characterizations and numerical studies to add knowledge on the topic.

Promising results concerning the significant enhancement in the mechanical (hardness and scratch resistance) and thermal properties (decomposition temperature) of a polyurethane (PU) filled with well-dispersed graphene oxide (GO) have been reported by Cai et al. [11]. Improvements in the storage modulus, thermal stability, and glass transition temperatures (*T_g_*) of nanocomposites based on a thermosetting epoxy resin and graphene nanoplatelets have been observed by Yasmin and Daniel [12]. Nanocomposites based on poly(ethylene terephthalate) (PET) and exfoliated graphite (EG) particles are prepared by melt-compounding method and then experimentally characterized in terms of thermal stability mechanical and electrical properties, which enhance significantly with the increment of EG concentration [13].

Polysulphone (PSU) nanocomposites with enhanced thermal properties due to the addition of different concentrations of carbon nanotubes or graphene nanoplatelets were successfully developed through the solution casting process by Irshad et al. [14].

Improvements in the thermomechanical and physicochemical properties of hytrel-based nanocomposites filled with small amounts of nanostructured fillers such as carbon nanotubes, graphene, and layered silicates were also observed in Pandei et al. [15].

The combined effect of the incorporation of graphite nanoplatelets and thermal treatment on mechanical and thermal properties of polyurethane copolymer (PUC)-based nanocomposites were investigated in Albozahid et al. [16].

Nowadays, polymer composites with high thermal conductivity are also investigated for solving specifically heat dissipation issues. The advent of a new fabrication process, i.e., additive manufacturing (AM), allows to design heat sinks in a broad variety of shapes and made with different materials. Due to their high thermal conductivity, aluminum, copper stainless steel, or titanium alloys have been classically adopted for such purposes. Challenges in reducing cost, weight, and devices size cannot be overcome with these materials since heat exchangers realized with them are almost always based on fin-and-tube or plate-fin structures. Instead, 3D printing opens the way for the design of heatsinks with more complex forms, which could be more effective for dissipating heat in a range of thermal applications as well as the use of environment-friendly materials [17].

Conventionally, heat sinks have been fabricated through the extrusion of metals, such as aluminum and copper, which are high density and, in particular, not biodegradable materials. From an environmental point of view, El-Dessouky and Ettouney have estimated that the energy required for the production of a unit of mass of polymer is significantly lower than such metals [18]. This means lower consumption of fossil fuels, as well as lower emission rates of greenhouse gases and air pollutants. Moreover, due to the low surface energy of polymers, these materials show an improved fouling resistance than metals, which reduces maintenance costs such as cleaning and pumping [19]. In addition, the low surface energy typical of the polymers allows ensuring long-term durability of dropwise condensation, which enhances the condensation heat transfer coefficient with respect to that of metals [20]. A remarkable review of the advantages of polymeric materials in heat transfer applications in comparison to metallic ones was presented by Marchetto et al. [21].

Yang et al. by combining biodegradable polybutylene succinate (PBS) and polylactic acid (PLA), have proposed a blend with high strength and toughness particularly suitable for AM technology, based on fused deposition modeling (FDM), where a thermoplastic filament is heated, extruded and then deposited on a building plate for creating the desired 3D structure [22].

In line with these premises, Timbs et al. have presented their experimental study on the thermal performance of 3D-printed heat sinks based on thermally conductive polymer composites [23]. Their results show as effective heat sinks with oblique fins present a lower thermal resistance and a better convective heat transfer in comparison to classical straight finned heat exchangers [23,24]. With the aim to improve thermal management aspects of GaN transistors, Gerges et al. have been investigated a 3D-printed polymer-based heat dissipator that may be suitable for reducing the weight and cost of the thermal device [25]. Wei et al. have presented the first studies on the interesting possibility to successfully implement a microfluidics heatsink fabricated using 3D printing for large die size and high-power applications [26]. Michalak et al. [27] have reported a polymer-based heat sink that lies on impingement-cooling principles and fabricated with another widely adopted AM technique, i.e., stereolithography (SLA).

Also, combined experimental and simulation studies have been proposed for evaluating the thermal dissipation performance of a metal-polymer composite heat sink with high processing efficiency, cost-effectiveness, and light weight, which are desired features in the field of heat exchanger [28].

In our previous study, the authors have presented their experimental results on the thermal behavior of some carbon-based nanocomposites and their first multiphysics simulation studies on two heat sinks based on pure PLA and PLA reinforced with GNPs, in view of their potential use in heat transfer applications [29]. Both the experimental and simulation studies were conducted with reference to the room temperature. Differently, the present work deals with the experimental measurement and numerical prediction of the effect of temperature on the thermophysical properties of 3D-printed nanocomposites, including two different GNPs concentrations (3 and 6 wt%). More in detail, four different temperature values ranging between 298.15 and 373.15 K have been selected to analyze the resulting effect on the thermal response in terms of thermal conductivity, thermal diffusivity, and specific heat capacity. All these properties were measured by the laser flash method [30] instead of the hot disk sensor adopted in our previous paper [29,31]. Pure PLA is assumed for performance comparison. A preliminary morphological analysis aimed to investigate the dispersion state of filler within the polymeric matrix and a mechanical investigation for shortly characterize the materials under test from a mechanical point of view were performed before focusing on thermal properties.

Multiscale modeling and simulation studies represent unique approaches to investigate the structure–property relationships of novel and advanced materials based on nanoscale particles [32]. The spatial arrangement of nanoparticles in block copolymers under applied mechanical pressure as well as how it can affect the overall phonon transport properties of the resulting structures are theoretically investigated in Dai et al. [33].

Therefore, this study is completed by a multiphysics simulation activity aimed at numerically analyzing the thermal behavior at the different investigated temperatures of these materials for their potential use as heat sinks.

## 2. Materials and Methods

The biodegradable polymeric matrix designated for the manufacture of the nanocomposites was Ingeo™ Biopolymer PLA-3D850 (Nature Works, Minnetonka, MN, USA) since it is specifically developed for realizing 3D printer monofilaments given its interesting printing features in terms of rapid crystallization, odor emission, warping or curling, adhesion to building bed and so on. A glass transition temperature (*T_g_*) of 55–60 °C and a peak melt temperature of 165–180 °C characterize this polymer.

Industrial graphene nanoplatelets, i.e., GNPs (TNIGNP from Times Nano, Chengdu, China) characterized by a purity >90 wt%, a number of layers less than 30, a lateral size ranging between 2 and 16 µm, a resistivity less than 15 Ω∙cm and a density of 2.2 g/cm^3^ were adopted for the production of nanocomposites investigated in the present study. The term industrial was assigned by the manufacturer (Times Nano, Chengdu, China) to indicate the large-scale production of such filler that allows containing its cost. Nevertheless, the authors have already tested in previous studies such carbon-based particles funding them suitable as reinforcement, given the remarkable overall physical properties of the resulting nanocomposites [29].

The two filler contents (3 and 6 wt%) have been selected according to our previous results [34]. In particular, it has been observed that the incorporation of 6 wt% of GNPs within the PLA polymer leads to best results from a nanomechanical point of view. Further, an increase in weight percentage of GNPs causes aggregation phenomena and, therefore, a not suitable filler dispersion, which in turn results in a worsening of mechanical properties, especially as regards the hardness and elasticity [34]. The concentration at 3 wt% was investigated as an intermediate point with respect to the maximum concentration and the unfilled matrix.

About the preparation method, nanocomposites pellets of PLA/GNPs were prepared by melt extrusion through a twin-screw extruder (COLLIN Teach-Line ZK25T, Maitenbeth, Germany), set to a screw speed of 40 rpm and at a temperature between 170 and 180 °C. Both matrix and filler were dried for 4 h at 80 °C in a vacuum oven before being used. Firstly, a masterbatch of 6 wt% of filler was extruded and then diluted in the right proportions with PLA through a second extrusion cycle to manufacturing nanocomposites with 3 wt% of GNPs.

Starting from these nanocomposites pellets, the filaments for 3D printing (FDM) with a diameter of 1.75 mm were manufactured by a single screw extruder in the temperature range 170–180 °C with a screw speed of 10 rpm.

Finally, parallelepiped test specimens with geometry 10 × 10 × 2 mm^3^ were prepared with a layer-to-layer deposition by using the FDM technique through a German RepRap X400 Pro 3D printer (German RepRap GmbH, Feldkirchen, Germany).

The following main printing settings have been adopted: nozzle temperature between 210 and 220 °C, a bed temperature of 65 °C, an extrusion speed of 17 mm/s, an infill density of 100%, and an external and internal infill pattern of type rectangular.

**Figure 1:**
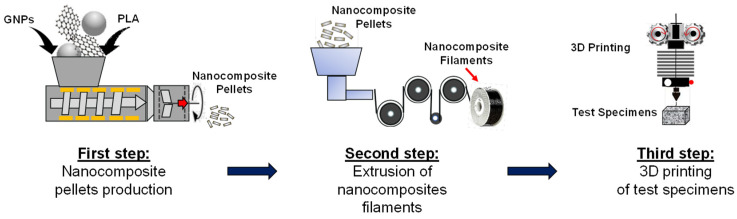
Schematic summary of the production of nanocomposites, filaments, and test samples.

All these manufacturing steps are schematically summarized in Figure 1.

Morphological study concerning the dispersion quality of graphene nanoplatelets into the PLA-based host matrix was explored through a scanning electron microscope (SEM) analysis performed with a field emission SEM apparatus JSM-6700F (JSM-6700F, Jeol, Akishima, Japan) on ad-hoc fractured in liquid nitrogen, etched and then gold-sputtered specimens as previously described in Spinelli et al. [29].

Since transmission electron microscopy (TEM) provides a higher resolution analysis compared to SEM, it is carried out for obtaining information about the filler size and aspect ratio. TEM micrograph of reinforced PLA is obtained through an FEI TECNAI G12 Spirit-Twin (LaB6 source, FEI Company, Hillsboro, OR, USA) operating with an acceleration voltage of 120 kV and a magnification ranging between 22 and 300 kX achievable with the support of an FEI Eagle-4k charged coupled device camera (CCD). By using a Leica EM UC6/FC6 ultramicrotome, appropriate sections for the 400 mesh TEM copper grids are obtained from the intact produced specimens.

Nanoindentation measurements were performed on the materials to investigate their micro-scale plastic features such as hardness and Young’s modulus based on the Oliver–Pharr model [35]. It is worth emphasizing that the considered nanoindentation Young’s modulus refers to Young’s modulus of elasticity received by the nanoindentation test using the above-mentioned method [35]. This is the elastic modulus related to the elastic displacement occurring in the specimen under investigation and not to the reduced modulus of elasticity, which is another interesting property investigated in the mechanical characterization of the polymers [36]. The Poisson’s ratio of polylactic acid-based composite materials has been laid down in the instrumental software prior to the calculation of the nanomechanical results. The authors have set it to the value of 0.36 as reported in the literature [37]. It should be mentioned that other values for Poisson’s ratio were examined, and no considerable change in the results was found. The tests were carried out using a Universal Nanomechanical Tester (UNMT, Bruker Surface Analysis, Minneapolis, MN, USA), equipped with a Berkovich Diamond indenter (Bruker, Minneapolis, MN, USA) with a tip radius of about 70 nm. All tests were performed in a force-controlled mode at a maximum force value of 100 mN.

For the tests, 48 nanoindentations have been scheduled according to an arrangement on a bidimensional array (4 × 12) with an indent separation of 80 µm.

Each one of these indentations consists of the following steps: (i) approaching the surface; (ii) loading to the peak load of 100 mN for 15 s; (iii) holding the indenter at peak load for 10 s; (iv) unloading from the maximum force of 100 mN to 10% for 15 s; (v) holding at 10% of max force 15 s; (vi) complete unloading for 1 s. Or, in other terms, a trapezoidal load function of 15-10-15 s was applied.

The tribological properties measurements (scratch and wear) were performed with a UMT–2M Universal Tester (UMT–2M, Bruker, Minneapolis, MN, USA). During the experimental measurement, the following parameters are continuously monitored and recorded: tangential force (*Fx*), normal load (*Fz*), and the coefficient of friction (*COF*). The last one is assessed by calculating the ratio between the tangential force and normal load (*COF = Fx/Fz*). A diamond tip with a radius of 0.4 mm and a normal load of 2 N was adopted for the scratch tests on the 3D-printed specimens, whereas their wear behaviors were investigated through a reciprocating motion test conducted by using a chrome stainless steel ball (radius of 3.18 mm) and a friction load of 2 N.

And finally, to conclude on the mechanical properties, flexural stress and tangent modulus of elasticity of the samples were evaluated, according to ASTM D790–07 (American Society for Testing and Materials) standard test methods, using a three-point loading system (1000 N force sensor) through a UMT–2M Universal Tester (UMT–2M, Bruker, Minneapolis, MN, USA).

**Figure 2:**
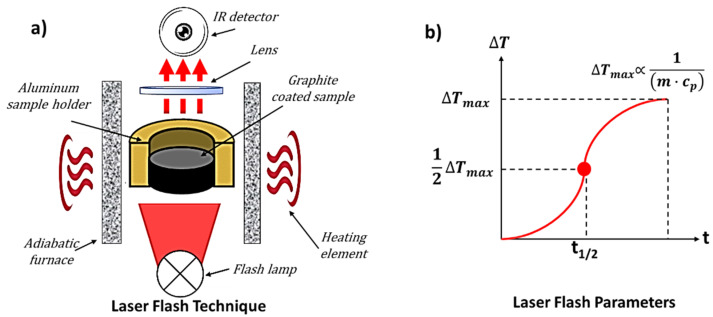
Measurement principle of laser flash method (**a**) and characteristic parameters for the evaluation of the thermophysical properties (**b**).

The laser flash analysis (LFA) or laser flash method is one of the most widely adopted techniques for measuring the thermal diffusivity of a great variety of sample materials. It was originally developed by Parker et al. in 1961 [30]. The basic principle is briefly reported below. During a measurement, an energy pulse with a Gaussian distribution in time heats the front face of a plane-parallel sample, whereas an infrared detector measures the resulting temperature change versus time on the rear surface of the same sample as schematically illustrated in Figure 2a. Duration and intensity of the pulse should be large enough to heat up, as uniform as possible, the backside face of the sample to around 1 K. The higher the thermal diffusivity of the test sample, the faster the temperature reaches the backside.

From the knowledge of the half time (*t*_1/2_, time value at half temperature course) as depicted in Figure 2b and sample thickness (*dz*), it is possible to derive the thermal diffusivity (*α*) and then the thermal conductivity (*λ*) as follows [30]:

(1) $$\alpha =0.1388\cdot \frac{dz^{2}}{t_{1/2}}$$

and

(2) $$\lambda \left(T\right)=\alpha \left(T\right)\cdot \rho \left(T\right)\cdot c_{p}\left(T\right)$$

where ρ(T) and $c_{p}\left(T\right)$ are the density (kg/m^3^) and the specific heat capacity (J/kg∙K) of the material, respectively, which in turn can be evaluated by comparing height (Δ*T_max_*) recorded for the sample with the signal height of reference material.

In the present study, the samples, approximately 10 × 10 mm with a thickness of 2 mm, were tested with the Laser Flash Technique (LFA 467 Hyper flash, Neztsch, Selb, Germany) at 4 different temperatures, 298.15, 318.15, 358.15, and 373.15 K. Each sample was measured five times at each temperature step. The tables of results report the average values.

These temperature values were chosen considering the value of the glass transition temperature for the pure PLA declared by the manufacturer, which is expected around 333.15 K. Such value is also confirmed by differential scanning calorimetry (DSC) measurements performed by the authors and reported in Batakliev et al. [38] An almost uniform discretization on four levels around this value is adopted. This is in light of a design of experiment (DoE) to be performed in future work with an approach already successfully applied in our previous studies [29,39,40]. This approach is particularly indicated in case of interest to statistically analyze the effect of some conditioning parameters on a selected performance function.

Prior to the measurements, the front and the back of the samples were coated with graphite to enhance the emission/absorption properties of the samples. The specific heat was determined by the reference method given by ASTM-E 1461-2011. Therefore, the LFA was calibrated with a Cp-standard (Pyroceram: squares with 10 mm in size and thickness of 2 mm). The density at room temperature was measured by using the buoyancy flotation method.

**Figure 3:**
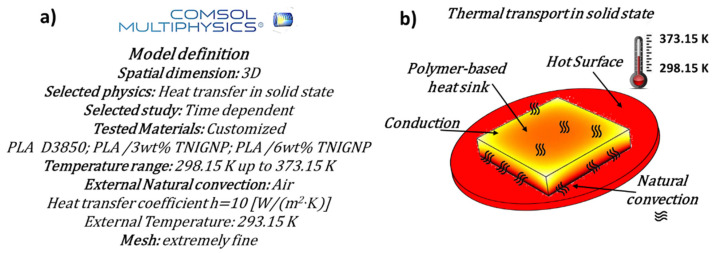
Main model definitions for the numerical study on the thermophysical properties of polymer-based heat sinks (**a**). Schematic representation of the numerical case study (**b**).

The applicability of advanced polymer-based heat sinks in heat transfer was numerically investigated through a 3D finite element simulation study performed with the commercial software COMSOL Multiphysics^®^ (version 5.5) whose main model definitions are collected in Figure 3a, whereas a schematic representation of the case study simulated in the present work, is reported in Figure 3b.

The experimentally tested samples are faithfully reproduced in a software environment where a square piece of 10 × 10 × 2 mm^3^ acts as a heat sink.

The thermophysical properties of the simulated heatsink are evaluated, as in a real practical application, placing its underside in contact with a hot surface at the different investigated temperatures (from 298.15 to 373.15 K) and observing the thermal response while it exchanges heat in still air at a room temperature of 293.15 K.

The heat is transferred inside the body of the heatsink by conduction and to the air around it through natural convection due to the density difference between the hot air adjacent to the hot surface and the colder air surrounding both the lateral and upper surfaces of the specimen (Figure 3b).

So that, it is possible to write the unsteady state heat balance in a cartesian coordinate on a differential volume *dx∙dy∙dz* as:

(3) $$-\left[\frac{\partial }{\partial x}\left(q_{x}\right)+\frac{\partial }{\partial y}\left(q_{y}\right)+\frac{\partial }{\partial z}\left(q_{z}\right)\right]=\rho \;c_{P}\frac{\partial T}{\partial t}$$

from which, considering the Fourier’s law of the heat conduction:

(4) q=−λ·∇T 

according to which the heat flux (*q*, (W/m^2^)) is proportional to the temperature negative gradient (∇*T*, (K/m)) through the thermal conductivity (*λ*, (W/(m∙K)), can be derived the differential equation of heat conduction:

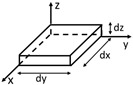
(5)$$\frac{\partial }{\partial x}\left(\lambda \frac{\partial T}{\partial x}\right)+\frac{\partial }{\partial y}\left(\lambda \frac{\partial T}{\partial y}\right)+\frac{\partial }{\partial z}\left(\lambda \frac{\partial T}{\partial z}\right)=\rho \cdot c_{P}\frac{\partial T}{\partial t}$$

As initial condition (*t* = 0), the heat sink is assumed to be at room temperature *T*_0_; as boundary conditions, the lower surface (*z* = 0) is in contact with the hot surface at temperature *Ts* (*Ts =* 298.15, 318.15, 358.15, and 373.15 K in the four simulated cases, respectively), whereas heat losses are considered at the lateral, upper, back and front surfaces given to the natural convection. All analytic terms concerning the initial and boundary conditions to correctly solve the thermal energy Equation (4) are reported in Table 1.

## 3. Results

### 3.1. Morphological Analysis

**Figure 4:**
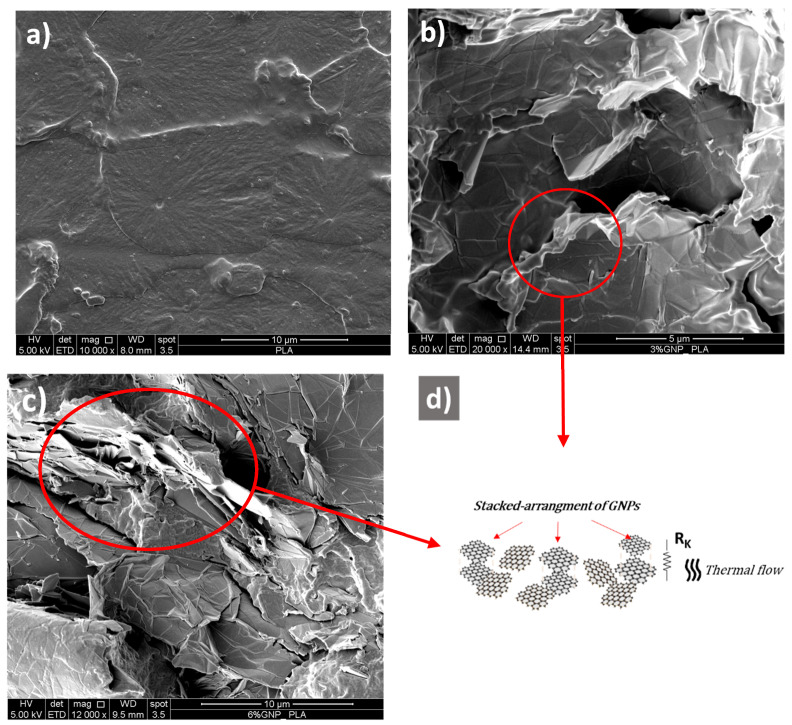
SEM images of neat PLA and the two nanocomposites, including 3 and 6 wt% of GNPs in (**a**–**c**), respectively. A schematic stacked arrangement of the graphene particles is depicted in (**d**).

**Figure 5:**
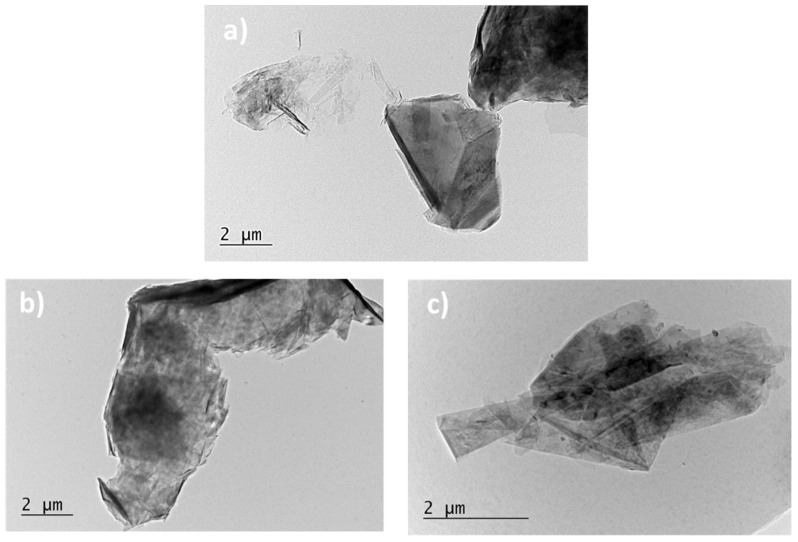
TEM images regarding the graphene nanoplatelets adopted in the present study as reinforcement for the PLA matric are reported in (**a**–**c**).

Scanning electron microscopy (SEM) and transmission electron microscopy (TEM) were performed, respectively, with the principal aim to investigate the morphology of the conductive phase (GNPs) and to evaluate their approximate size. Figure 4 depict the SEM images concerning the pure polymer (PLA) in (a), the nanocomposite with 3 wt% of GNPs in (b), the nanocomposite based on 6 wt% of GNPs in (c), whereas Figure 5 shows different TEM images focused on the adopted graphene nanoplatelets in (a), (b), and (c).

With reference to the unfilled polymer (Figure 4a), it is evident as the surface appears quite flat with a reduced effect of roughness and graininess compatible with the fracturing treatment through liquid nitrogen. This expedient determines a brittle fracture without deformation prior to failure that typically occurs with alternative approaches. From the SEM images regarding the nanocomposites with 3 and 6 wt% (Figure 4b,c, respectively), it is worth noting in both cases some cavities due to chemical etching applied to the specimens prior to morphological investigation.

In addition, an even more important aspect, it is evident that a suitable dispersion of the GNPs particles in a sort of a stacked arrangement that acts as a thermally conductive pathway with a lower graphene-graphene contact resistance, which is suited for a more efficient phononic transport (Figure 4d). This last is also favored by the easiest wetting of bidimensional surfaces of GNPs by the polymer. As a result, the interfacial thermal resistance, also known as Kapitza resistance (R_k_), is reduced, and the heat flow is enhanced [41].

A structural analysis has shown how the dispersion method of graphene nanoplatelets into a polymer matrix can strongly affect the GNPs morphology [42]. It is important to point out that the as-received filler is already in a stacked form, which is preserved during the extrusion process. Moreover, this arrangement is also favored by the layer-by-layer sample production through 3D printing technology based on fused deposition modeling (FDM), as already observed with other layer-by-layer growth mechanisms and nanoparticles [43].

And finally, for concluding the morphological investigation, the TEM micrographs of Figure 5a–c confirm the planar rectangular structure of the filler with an average size that falls in the interval 5–10 µm, in agreement with the technical specification provided by the manufacturer.

### 3.2. Mechanical Properties as Function of GNPs Content

Prior to thermally investigating the samples, a preliminary mechanical analysis has been performed to briefly characterize such materials in terms of mechanical properties.

In prospective of their potential use as heat sinks, such characterization is important since it is necessary to consider that every one device could be damaged during removal, transportation, or unintentionally throughout the exploitation period. In addition, when a new material is offered as a replacement for an existing one for the respective application, it is important that its mechanical properties are close to those classically used so far.

**Figure 6:**
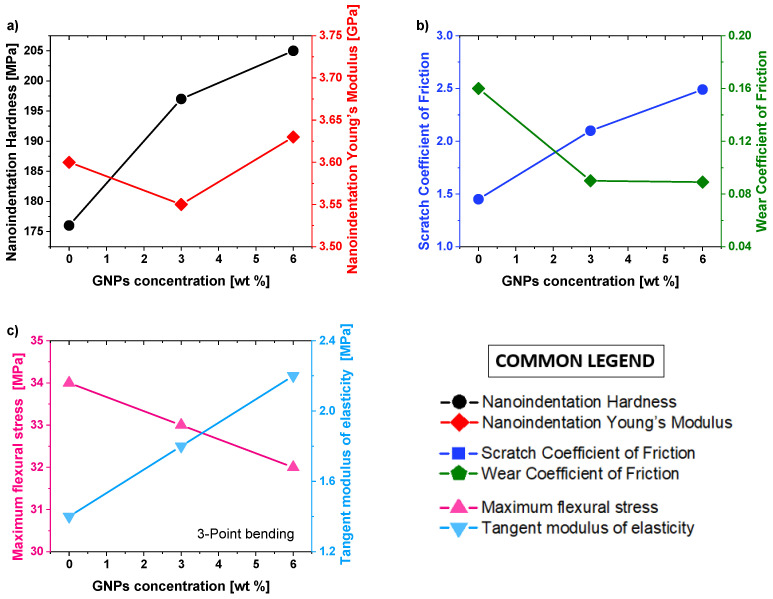
Comparison of mechanical properties of pure PLA, PLA filled with 3 wt% GNP and PLA reinforced with 6 wt% GNP in terms of nanoindentation hardness and Young’s modulus in (**a**), scratch and wear coefficient of friction in (**b**), maximum flexural stress and tangent modulus of elasticity in (**c**).

Figure 6a–c reports the results concerning nanoindentation hardness and Young’s modulus in (a), scratch and wear coefficient of friction in (b), maximum flexural stress, and tangent modulus of elasticity in (c) as a function of the GNPs filler content.

With reference to Figure 6a, as expected, the maximum value of nanoindentation hardness (left axis, black line, and markers) is reached at 6 wt% of GNPs, where the improvement of such mechanical property due to the introduction of this strong reinforcement is about 16.5% compared to the value exhibited by the unfilled PLA. Differently, the values for Young’s modulus (right axis, red line, and markers) slightly change with the increase in the filler loading due to the exfoliation degree of the GNPs in the polymer matrix.

The low value of nanoindentation Young’s modulus, ascertained for the composite 3 wt% GNP/PLA, is most likely due to a lower level of graphene’s exfoliation in the elastic zone of the specimen.

The friction coefficient is a parameter of great interest when it comes to the mechanical properties of nanomaterials since it provides information on surface damages after tribological treatment. Different from the classical indentation process where a normal load is uniformly applied through the indenter, a scratch test is based on a high-friction-induced sliding phenomenon. As a result, it is possible to evaluate the material resistance to mechanical surface damages under this process.

The results shown in Figure 6b (left axis, blue line, and markers) reveal that the scratch resistance of the samples increases with the progressive addition of GNPs. This effect reaches a maximum at 6 wt% GNP, where the value of *COF* is 2.49 compared with 1.45 measured for the pure PLA matrix, which corresponds to about 72% of improvement for the coefficient of friction at scratch. Most likely, it is due to the enhanced tangential force (*Fx*) in nano-reinforced structures, which in turn leads to higher *COF,* as also observed by Porwal et al. [44].

The results depicted in Figure 6b show the incorporation of GNPs in the polymer matrix reduces and then improves the wear coefficient of friction (right axis, green line, and markers) of the composites in general, as also observed in the experimental study of Bustillos et al. [45]. This can be explained by the self-lubricating effect of GNPs incorporated in the PLA matrix, which helps to reduce the friction and wear of the nanocomposites. This effect is more pronounced at 6 wt% GNP, where the maximum improvement is about 80%.

For the sake of completeness, it is worth reporting that, by means of an in situ scanning probe microscopy (SPM) imaging reported in Batakliev et al. [46], an average surface roughness (Ra) in the range of 4–11 nm was observed for these 3D-printed samples, which is suitable for the performed mechanical tests.

Finally, Figure 6c presents the results for maximum flexural stress (left axis, pink line, and markers) and tangent modulus of elasticity (right axis, light blue line, and markers) measured during the three-point bending tests of the nanocomposites PLA/3 wt% GNPs, PLA/6 wt% GNPs) and pure PLA for comparison. It is possible to note how the maximum flexural stress slightly decreases with the increasing of the GNPs due to the poor dispersion and specific geometry of the nanofiller, which, however, leads to the improvement of the tangent modulus of elasticity of about 57% at 6 wt% GNPs.

All the results of this mechanical characterization are summarized in Table 2.

### 3.3. Thermophysical Properties as Function of Temperature

**Figure 7:**
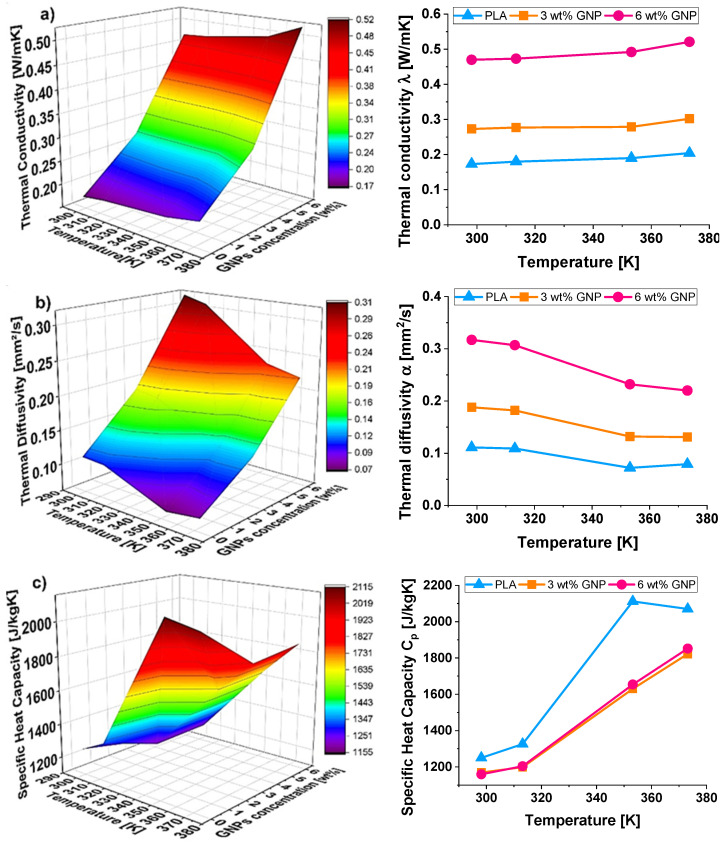
Comparison of thermophysical properties of pure PLA, PLA filled with 3 wt% GNPs and PLA reinforced with 6 wt% GNPs in terms of thermal conductivity (*λ*), thermal diffusivity (*α*), and specific heat capacity (Cp) in (**a**–**c**), respectively.

Figure 7 from (a) to (c) (3D view on the left and 2D graphics on the right) depict the thermal conductivity (*λ*), the thermal diffusivity (*α*), and specific heat capacity (*C_p_*) as a function of GNPs content and of the temperature in the range between 298.15 and 373.15 K. More in detail, Figure 7a shows the comparison of the thermal conductivity of all samples. A clear increase in thermal conductivity with increasing filler content is observed. In fact, at the temperature of 298.15 K, the thermal conductivity for the pure PLA is 0.173 W/mK, whereas for PLA with 6 wt% of GNPs reaches the value of 0.470 W/mK, which corresponds to a significant improvement of about 171%.

Therefore, since classical thermally insulating materials show a thermal conductivity of the order of 10^−3^ (W/m K), the proposed nanocomposites, in light of the measured values of conductivity and in combination with the other benefits typical of polymer materials, can be considered suitable for potential heat transfer applications.

With reference to the temperature influence, the thermal conductivity increases slightly as it increases, at least in the investigated temperature range. This is because, with increasing temperature, the molecular vibrations increase, thus leading to a higher phonon propagation and hence to a higher thermal conductivity [47,48].

However, the temperature has a great impact on the thermal conductivity of nano-reinforced polymers due to different mechanisms such as scattering mechanism, changes in specific heat, polymer chain orientation, and so on. In brief, up to a certain temperature, these influencing parameters favor the thermal conductivity, which will start to progressively increase with the temperature. Differently, at higher temperatures, mainly due to the anharmonic scattering and changes in the molecular morphology, a balance (or also a worsening) between phonon propagation and its scattering is reached and, consequently, it is expected that the thermal conductivity reduces with temperature [49,50].

Differently, as can be noted from Figure 7b, the thermal diffusivity decreases with increasing temperature. In particular, the change in slope between 313.15 and 353.15 K is caused by the glass transition (which is expected around 333.15 K). The glass transition is also indicated in the specific heat determination by a step (Figure 7c).

By comparing these trends all together (Figure 7a–c), it is interesting to note how the temperature dependence for the conductivity, at least in the investigated range, presents a sweet profile that becomes slightly more marked for diffusivity, whereas it is clearly evident for the specific heat capacity.

Table 3, Table 4 and Table 5 summarize the corresponding measurement data.

It is worth pointing out that the experimental evaluation of the specific heat capacity was obtained by neglecting the temperature dependence of the density. This could affect the measurement, especially at higher temperatures. Nevertheless, the experimental results reported both in Figure 7c and in Table 3, Table 4 and Table 5 are well interpolated with a polynomial regression of the second order, according to the literature [51]. The following equations and related regression coefficients (*R*^2^) are obtained:

(6) $$\mathrm{pure}\;\mathrm{PLA}:\;C_{p}=-0.1161T^{2}+90.723T-15555\;;\;R^{2}=0.935$$

(7) $$\mathrm{PLA}/3\;\mathrm{wt}\%\;\mathrm{GNPs}:\;C_{p}=0.0511T^{2}-25.089T+4081.1;\;R^{2}=0.989$$

(8) $$\mathrm{PLA}/6\;\mathrm{wt}\%\;\mathrm{GNPs}:\;C_{p}=0.0452T^{2}-20.607T+3262.2;\;R^{2}=0.991$$

### 3.4. Simulation Studies of Thermophysical Properties

**Figure 8:**
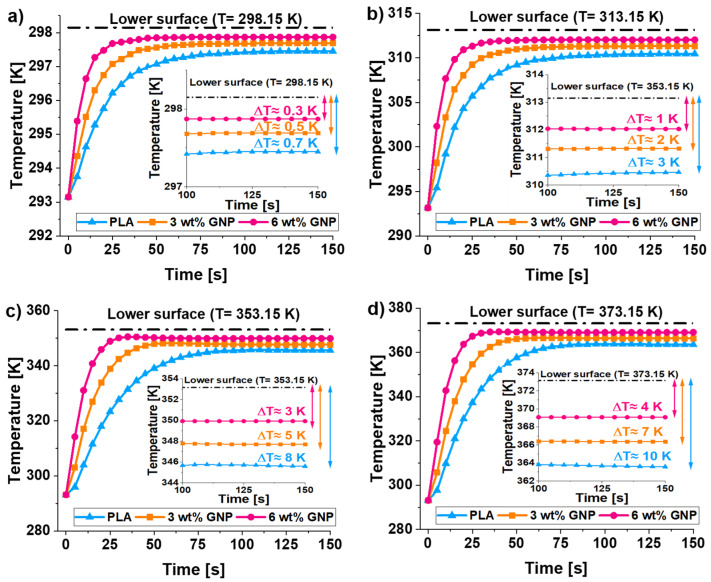
Comparison of the temperature profiles (average values) recorded on the upper surfaces vs. time for heat sink based on pure PLA, PLA reinforced with 3 and 6 wt% of GNPs different temperatures, i.e., 298.15, 313.15, 353.15, and 373.15 K, are considered in (**a**–**d**), respectively.

Figure 8 reports the temperature profiles (average values) recorded on the face opposite to that in contact with the hot surface as a result of the combination of conductive and convective transport. In particular, the thermal responses at the different simulated temperatures, i.e., 298.15, 313.15, 353.15, and 373.15 K, are shown from Figure 8a–d, respectively.

It is interesting to note as, regardless of the temperature value, the less thermally conductive composite (pure PLA) has a more pronounced temperature gap (∆*T*) between the lower and upper surfaces, which progressively reduces with the increasing of the concentration of GNPs according to a progressive improvement of the thermal conductivity (*λ*) of resulting material.

Furthermore, from the analysis of these results, it is evident as the higher the initial temperature, the greater this gap. At 298.15 K, ∆*T* results of about 0.3, 0.5, and 0.7 K for unfilled PLA and for that filled with 3 and 6 wt%, respectively. At 373.15 K, these values become 4, 7, and 10 K, respectively.

Then, again, it is possible to observe that, regardless of the starting temperature value, during the thermal transient, the temperature on each upper surface increases as quickly as more thermally conductive is the material due to the greater internal heat flux.

**Figure 9:**
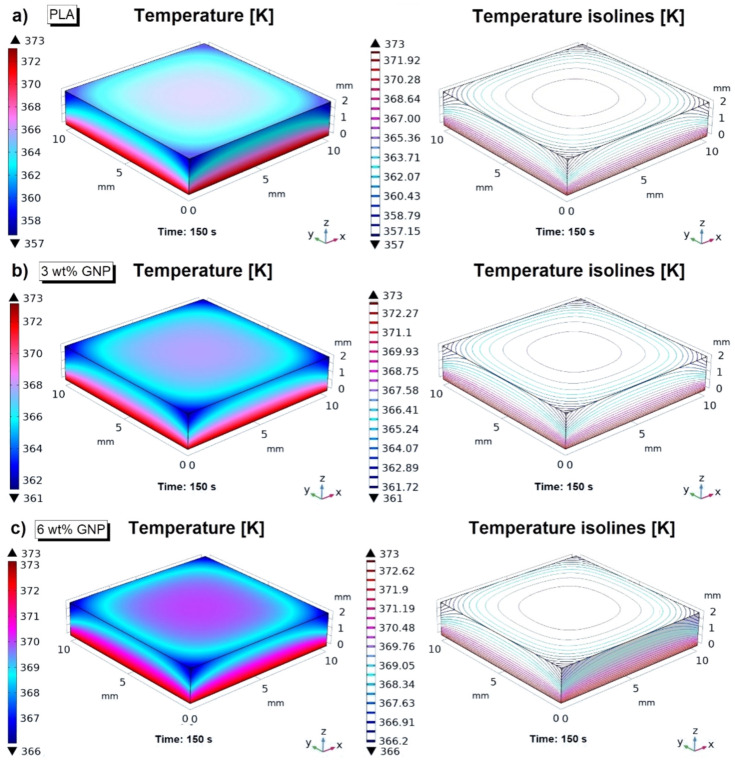
Numerical prediction of the average surface temperature profiles (**left side**) and temperature isolines (**right side**) recorded at steady-state condition (*t* = 150 s), and at temperature value of 373.15 K for heat sinks based on pure PLA, PLA reinforced with 3 wt% of GNPs and 6 wt% of GNPs in (**a**–**c**), respectively.

In order to deepen this argument, Figure 9 takes into account the 3D average surface temperature profiles (left parts) and the recorded temperature isolines (right side) estimated for the heat exchangers (pure PLA, PLA filled with 3 and 6 wt% in (a), (b), and (c)) at steady-state condition (*t* = 150 s) and at the highest investigated temperature value (373.15 K).

Looking at these graphs, the various tones of colors best represent the different thermal response behavior of the simulated polymer-based heat sinks. In the case of 6 wt% of GNPs, the temperature appears more evenly distributed in all axial directions, whereas a more pronounced thermal gradient can be noted as the GNPs content decreases.

With reference to Figure 9c, it is interesting to note as the heat transfers, due to the higher thermal conductivity, through the solid going to warm its top surface. Differently, for the pure PLA (Figure 9a), since it is characterized by a lower thermal conductivity, the internal conductive transport is limited, and therefore, the upper surface remains noticeably colder with respect to its warmer opposite face. Intermediate behavior is observed for PLA filled with 3 wt% of GNPs (Figure 9b). Moreover, the comparison between the temperature isolines indicates a more uniform heat distribution, especially on the top surface, for the nanocomposite with the highest filler content (Figure 9c) deductible from its larger isolines than those belonging to 3 wt% of GNPs and pure PLA.

Instead, regardless of the heat sink type, a remarkable edge effect is present because of the heat exchange with the environment (natural convection), which is carefully considered in this simulation study. As a result of this thermal transfer associated with the cooling in still air at a temperature of 293.15 K, the temperature at the edges as well as at the upper corners of the exchangers is decisively lower than anywhere else in the solid.

**Figure 10:**
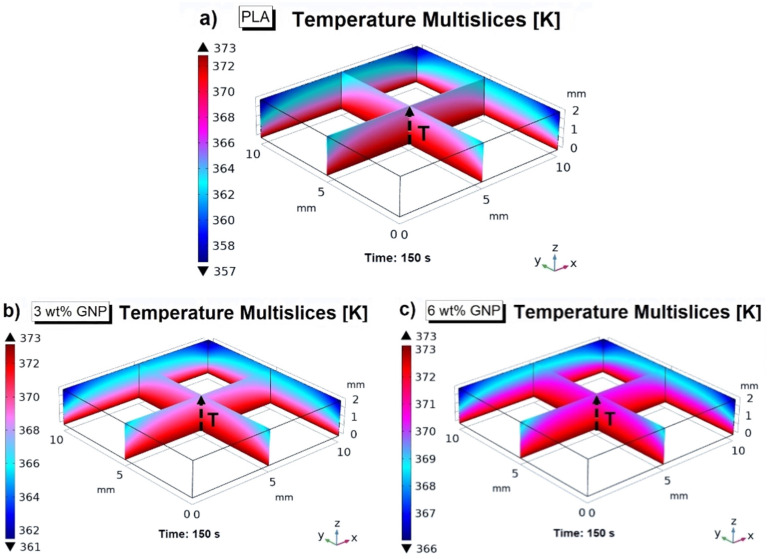
Temperature multislices evaluated at steady-state condition (*t* = 150 s) and at temperature value of 373.15 K relatively to PLA, PLA/3 wt% GNPs, and PLA/6 wt% GNPs in (**a**–**c**), respectively.

This effect results in more appreciable with reference to the temperature multislices representations reported in Figure 10, which are always revealed at steady-state condition (*t* = 150 s) and at the highest investigated temperature value (373.15 K). The case of unfilled PLA, PLA reinforced with 3 wt% of GNPs and 6 wt% of GNPs, is examined in Figure 10a–c, respectively. In particular, these graphical views allow us to note both as, in general, the temperature is more concentrated in the lower and internal parts of the solid due to the reduced or absent external heat exchange and as the temperature is better distributed in the case of 6 wt% of GNPs given its higher thermal conductivity that favors the thermal transport within the medium, respect the other investigated cases.

For continuing the thermal analysis, in the same Figure 10, at the intersection of the two central slides, there is a dashed arrow that runs through the entire thickness of the heat sink.

**Figure 11:**
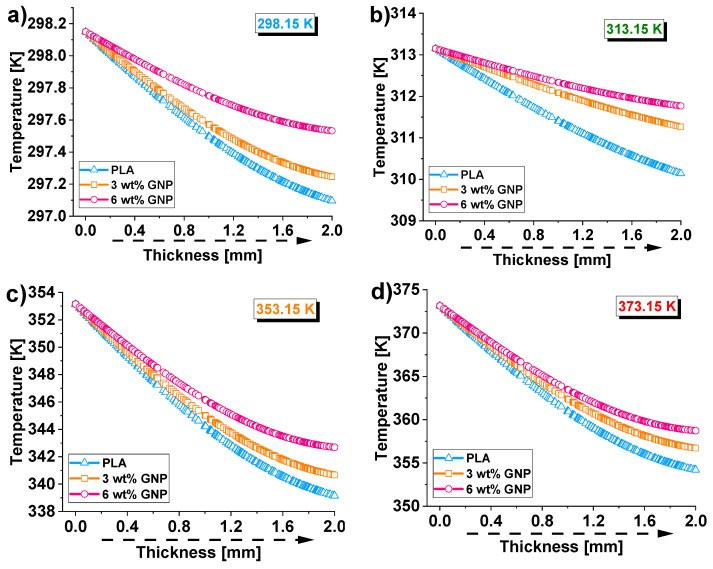
Temperature profiles (evaluated at the half course of each transient phase and alongside the symmetry axis) for the different heat sinks at the different temperature values: 298.15, 313.15, 353.15, and 373.15 K in (**a**–**d**), respectively.

Along this direction, the next Figure 11 shows the thermal profiles evaluated at the half course of each transient phase and for all temperatures under examination, i.e., 298.15, 313.15, 353.15, and 373.15 K in Figure 11a–d, respectively.

By analyzing these curves, it is possible to notice the expected decrease in temperature along the thickness as it moves far from the lower surface (*z* = 0) up to the top one (*z* = 2).

In particular, at low temperatures (298.15 K and 313.15 K), this decreasing trend is quite linear, at least up to the mid-thickness. Otherwise (353.15 K and 373.15 K), it does not appear so linear.

Moreover, also from these graphics, it is worth noting that the higher the thermal conductivity of the material, the lower is the temperature gap (∆*T*) along the thickness, as previously discussed.

These results are due not only to the different internal conductive flow linked to the respective intrinsic conductivity values but also to the different heat exchanges with the surrounding environment, given the different surface temperature values.

**Figure 12:**
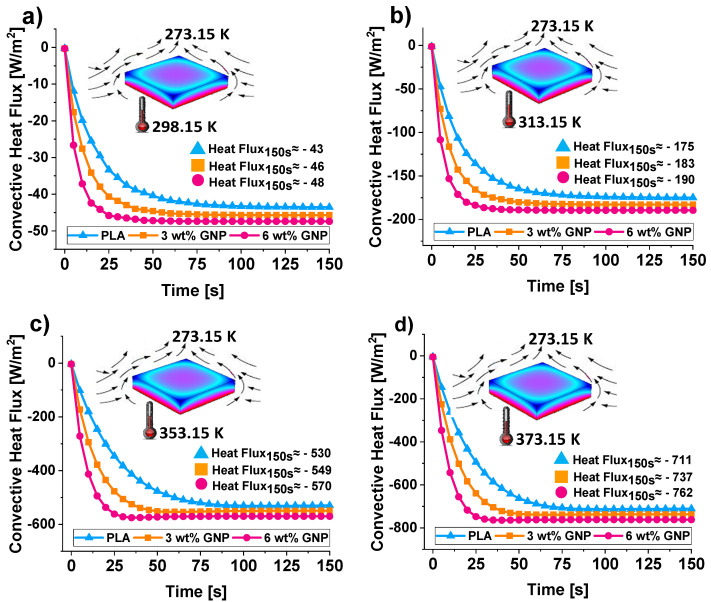
Convective heat flux trends over time for the three polymer-based heat exchanges operating at different temperature values: 298.15, 313.15, 353.15, and 373.15 K in (**a**–**d**), respectively.

In light of this, Figure 12 from (a) to (d) shows the convective heat flux trends (average values) by natural convection versus the time at different selected temperatures.

During the transient phases, for each temperature, the convective fluxes seem to be evenly spaced.

At the end of simulation time (150 s), in the steady-state region and at a temperature of 298.15 K, a value of −43 (W/m^2^), −46 (W/m^2^), and −48 (W/m^2^) are predicted for the conductive heat flux for PLA, PLA/3 wt% of GNPs, PLA/6 wt% of GNPs, respectively.

At the highest analyzed temperature (373.15 K) such values turn in −711 (W/m^2^), −737 (W/m^2^), and −762 (W/m^2^), respectively.

The larger value (in modulus) for the convective flux exhibited by the nanocomposite with the highest GNPs concentration (6 wt%) indicates a greater efficiency of this polymer-based heat exchanger in dissipating heat to the surrounding air, and then it can be elected the most indicated, among those assessed in the present work, for practical thermal applications.

**Figure 13:**
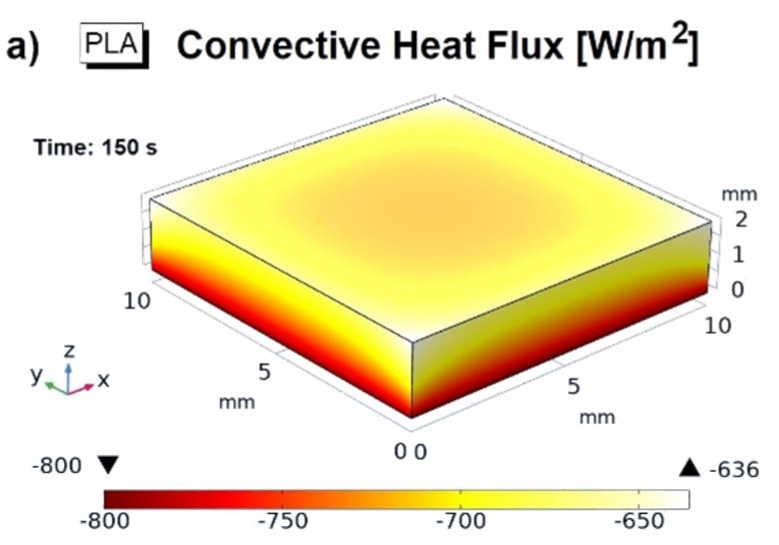

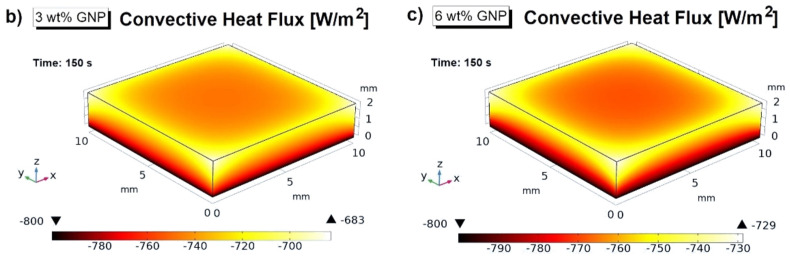
Three-dimensional views of the conductive heat flux at steady-state condition (*t* = 150 s) and at temperature value of 373.15 K relatively to PLA, PLA/3 wt% GNPs, and PLA/6 wt% GNPs in (**a**–**c**), respectively.

A 3D view of the conductive heat flux at steady-state condition (*t* = 150 s) and at a temperature value of 373.15 K relatively to PLA, PLA/3 wt% GNPs, and PLA/6 wt% GNPs are shown in Figure 13a–c, respectively. Suh graphics allow to better distinguish the different connective flux rates, at the solid/air exchange surface, between the pure and reinforced PLA. The top surface of the heat sink is the most involved in this thermal exchange with the surrounding air due to its greater exchange surface than the other sides. However, the maximum heat transfer is recorded in the down sides of the heatsink, given the higher temperature values in such area. A more efficient convective heat flux is noted for the highest filled nanocomposite, i.e., 6 wt% of GNPs.

**Figure 14:**
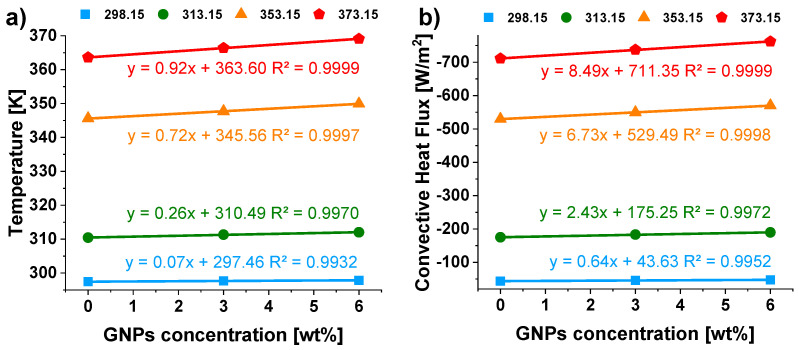
Temperature (**a**) and convective heat flux (**b**) as a function of GNPs concentration at the different temperatures. The lines are the fitting curves of the numerical data (colored markers).

Finally, Figure 14 show as both the average temperature on the upper surface of the heat sinks (Figure 14a) and the convective flux (Figure 14b) vary, regardless of the temperature value, in a perfectly linear way (the coefficient of determination *R*^2^ strictly close to 1 for each fitting curve) with the GNPs concentration.

Such statistical results might be useful for further thermal predictions as the first approach in evaluating the expected thermal behavior of these novel materials.

## 4. Discussion

The effect of temperature on the thermophysical properties of poly(lactic acid) (PLA) reinforced with two weight percentages (3 and 6 wt%) of graphene nanoplatelets (GNPs) has been experimentally and numerically analyzed. A preliminary morphological analysis has shown a suitable dispersion of the carbonaceous filler within the polymeric matrix as well as their arrangement in a sort of stacked structure, which is favorable for enhancing the thermal transport due to the consequent reduction in the interfacial (matrix/filler) and interparticle thermal resistance.

Furthermore, a preliminary mechanical characterization has been carried out to briefly characterize such samples from a structural point of view. It is found that, thanks to the addition of 6 wt% of GNPs, the nanoindentation hardness of the resulting material is improved by about 16.5% compared to the value exhibited by the pure PLA. This result can be assigned to the remarkable intrinsic mechanical features of the strong reinforcement adopted in the present study. In addition, the scratch resistance of the samples is enhanced with the addition of GNPs. Always at the 6 wt% GNP, the value of the coefficient of friction (COF) is a result of 2.49, whereas the value of 1.45 is measured for the unfilled matrix, which corresponds to about 72% of improvement for this mechanical property. Most likely, it is due to the enhanced tangential force (*Fx*) in nano-reinforced structures, which in turn leads to higher *COF,* as also observed in other literature studies.

About the thermal behavior of the designed nanocomposites, the effect of temperature has been investigated in the temperature range from 298.15 to 373.15 K. The most performing nanocomposite (6 wt% of GNPs) has presented, at the lowest temperature (298.15 K), an improvement of 171% for the thermal conductivity respect to the PLA whereas at the highest temperature (372.15 K) such enhancement is resulted about of 155%. The reason is due to the contribution of graphene nanoparticles in reducing the thermal resistance (Kapitsa resistance), which, in turn, improves the thermal transport.

Comparable amounts of graphene nanoplatelets, such as those of the present study, led to the significant improvement of the thermal conductivity of nanocomposites based on other thermoplastic polymers, such as polycarbonate (PC) [52], or thermosetting ones, such as epoxy resins [53,54].

Regarding the temperature influence, it is experimentally observed that the thermal conductivity increases slightly as it increases, at least in the considered temperature range.

In fact, as expected from theory [47,48], the thermal conductivity of polymer composites based on thermally and electrically conductive particles increases progressively with temperature until they reach a maximum and then begin to fall. This because, with increasing temperature, the molecular and structural vibrations increase, thus leading to a higher phonon propagation and hence to a higher thermal conductivity, which will reach a maximum at certain temperatures. After that, with the increasing temperature and consequential vibrations, a balance between phonon transport and its scattering will be reached, and beyond this temperature, the thermal conductivity starts to reduce. Moreover, it is necessary to also consider that in conductive solids, heat can be conducted through two different mechanisms: the main is the lattice vibrations, and the second one is due to the free electrons flow, which can support the diffusion process of heat energy through the medium under specific conditions. As the solid heats up, the random collisions of electrons increase, thus hindering the flow of the phonons that, in fact, must compete with the behavior of electrons. It is essentially the same reason responsible for the electrical resistance increment with temperature observed in conductive solid.

Instead, near the glass transition (expected at ~333 K), it is observed that the thermal diffusivity decreases with increasing temperature.

In future work, the authors reserve to experimentally determine the value of the specific heat by means of a different method (for example, DSC) to consider the dependence of density on temperature.

A simulation study based on the finite element method (FEM) has been performed to numerically investigate the thermal behavior of the designed nanocomposites in prospective of their potential use in heat transfer application as polymer-based heat sinks. Different thermal profiles, as well as convective fluxes, have been carefully examined at the different temperatures selected for the experimental characterization. Once again, the numerical activity has shown that the heat exchanger realized with the highest filler concentration (6 wt% of GNPs) exhibit a greater efficiency in dissipating heat to the surrounding air, a more uniform internal temperature distribution and therefore it can be identified as the most promising, among those evaluated in the present work, for practical thermal applications.

## 5. Conclusions

This paper deals with the experimental and numerical study of the effect of temperature on the thermophysical properties of PLA-based composites reinforced with two concentrations of graphene nanoplatelets (GNPs). The recent additive manufacturing (AM) fabrication process has been used to produce the specimens.

Carbon-based nanofillers, when dispersed within polymeric matrices allow to significantly improve the thermal conductivity of the resulting nanocomposites. Therefore, it is motivated their use as heat sinks to dissipate heat in a wide range of thermal applications.

Since the topic aroused strong interest both in the academic and industrial community, a future paper will concern heat sinks with a more complex shape (thanks to 3D printing technology) and probably more suitable to overcome overheating effects, as well as other nanocomposite formulations, will be considered.

## Figures and Tables

**Table 1:** Initial and boundary conditions for solving the thermal energy equation.

Initial (I.C.) and Boundary (B.C.) Conditions		Equations	Validity
I. C.	*t* = 0	*T* = Room Temperature (*T*_0_)	∀x,∀y,∀z
B. C.	*Lower Surface* *z* = 0	T=Ts	(∀x,∀y,t>0)
B. C.	*Upper Surface* *z* = 2	$-\lambda \frac{\partial T}{\partial z}=h\cdot \left(T-T_{\infty }\right)$	(∀x,∀y,t>0)
B. C.	*Lateral Surfaces* *y* = 0 *y* = 10	$-\lambda \frac{\partial T}{\partial y}=h\cdot \left(T-T_{\infty }\right)$	(∀x,∀z,t>0)
B. C.	*Back and Front Surfaces* *x* = 0 *x* = 10	$-\lambda \frac{\partial T}{\partial x}=h\cdot \left(T-T_{\infty }\right)$	(∀y,∀z,t>0)

**Table 2:** Mechanical properties of the investigated nanocomposites.

Materials	Nanoindentation Hardness (MPa)	Nanoindentation Young’s Modulus (GPa)	Scratch Coefficient of Friction	Wear Coefficient of Friction	Maximum Flexural Stress * (MPa)	Tangent Modulus of Elasticity * (MPa)
PLA	176	3.60	1.45	0.16	34	1.4
PLA/3 wt% GNP	197	3.55	2.10	0.09	33	1.8
PLA/6 wt% GNP	205	3.63	2.49	0.089	32	2.2

* Test performed in 3-point bending configuration.

**Table 3:** Thermophysical properties of pure PLA ^1^.

Temperature (K)	Thermal Conductivity (W/mk)	Thermal Diffusivity (mm^2^/s)	Specific Heat Capacity (J/kgK)
298.15	0.173	0.111	1250
313.15	0.180	0.109	1326
353.15	0.190	0.072	2112
373.15	0.204	0.079	2070

^1^ Density at room temperature (20 °C): 1250 (kg/m^3^).

**Table 4:** Thermophysical properties of PLA/3 wt% GNPs ^1^.

Temperature (K)	Thermal Conductivity (W/mk)	Thermal Diffusivity (mm^2^/s)	Specific Heat Capacity (J/kgK)
298.15	0.273	0.188	1169
313.15	0.277	0.182	1198
353.15	0.279	0.132	1630
373.15	0.302	0.131	1822

^1^ Density at room temperature (20 °C): 1270 (kg/m^3^).

**Table 5:** Thermophysical properties of PLA/6 wt% GNPs ^1^.

Temperature (K)	Thermal Conductivity (W/mk)	Thermal Diffusivity (mm^2^/s)	Specific Heat Capacity (J/kgK)
298.15	0.470	0.317	1159
313.15	0.473	0.307	1204
353.15	0.492	0.232	1654
373.15	0.521	0.220	1852

^1^ Density at room temperature (20 °C): 1280 (kg/m^3^).

## Data Availability

All data are contained within the article.
